# Ischemic preconditioning accelerates muscle deoxygenation dynamics and enhances exercise endurance during the work-to-work test

**DOI:** 10.14814/phy2.12395

**Published:** 2015-05-07

**Authors:** Kohei Kido, Tadashi Suga, Daichi Tanaka, Toyoyuki Honjo, Toshiyuki Homma, Satoshi Fujita, Takafumi Hamaoka, Tadao Isaka

**Affiliations:** Faculty of Sport and Health Science, Ritsumeikan UniversityKusatsu, Shiga, Japan

**Keywords:** Exercise, mitochondria, near-infrared spectroscopy, nitric oxide, skeletal muscle

## Abstract

Ischemic preconditioning (IPC) improves maximal exercise performance. However, the potential mechanism(s) underlying the beneficial effects of IPC remain unknown. The dynamics of pulmonary oxygen uptake (VO_2_) and muscle deoxygenation during exercise is frequently used for assessing O_2_ supply and extraction. Thus, this study examined the effects of IPC on systemic and local O_2_ dynamics during the incremental step transitions from low- to moderate- and from moderate- to severe-intensity exercise. Fifteen healthy, male subjects were instructed to perform the work-to-work cycling exercise test, which was preceded by the control (no occlusion) or IPC (3 × 5 min, bilateral leg occlusion at >300 mmHg) treatments. The work-to-work test was performed by gradually increasing the exercise intensity as follows: low intensity at 30 W for 3 min, moderate intensity at 90% of the gas exchange threshold (GET) for 4 min, and severe intensity at 70% of the difference between the GET and VO_2_ peak until exhaustion. During the exercise test, the breath-by-breath pulmonary VO_2_ and near-infrared spectroscopy-derived muscle deoxygenation were continuously recorded. Exercise endurance during severe-intensity exercise was significantly enhanced by IPC. There were no significant differences in pulmonary VO_2_ dynamics between treatments. In contrast, muscle deoxygenation dynamics in the step transition from low- to moderate-intensity was significantly faster in IPC than in CON (27.2 ± 2.9 vs. 19.8 ± 0.9 sec, *P* < 0.05). The present findings showed that IPC accelerated muscle deoxygenation dynamics in moderate-intensity exercise and enhanced severe-intensity exercise endurance during work-to-work test. The IPC-induced effects may result from mitochondrial activation in skeletal muscle, as indicated by the accelerated O_2_ extraction.

## Introduction

Ischemic preconditioning (IPC) by brief episodes of ischemia and reperfusion in the organs, especially the heart, provides protection from tissue damage such as myocardial injury (Murry et al. 1986). In the clinical setting, IPC is becoming an effective strategy for reducing the risk of myocardial injury and for improving the prognosis of patients after cardiac surgery (Thielmann et al. 2013). Alternatively, recent studies found that IPC can enhance endurance exercise performance (de Groot et al. 2010; Crisafulli et al. 2011; Bailey et al. 2012a,b). However, the potential mechanism(s) underlying the beneficial effects of IPC remain unknown.

Exercise performance is determined multifactorially, and oxidative metabolism is a major determinant. The dynamics of pulmonary oxygen uptake (VO_2_) and muscle deoxygenation during exercise are frequently used for assessing O_2_ supply and extraction (DeLorey et al. 2003; Wilkerson et al. 2004; Bailey et al. 2010). Those systemic and local O_2_ dynamics may be useful for understanding the beneficial effects of IPC on exercise performance. For example, the faster inductions of systemic and local O_2_ dynamics can be achieved using prior exercise, and these responses are closely related to cardiovascular and skeletal muscle activation (Burnley et al. 2000; Gurd et al. 2005; Marles et al. 2007; Faisal et al. 2009). Similarly, IPC also ameliorates the O_2_ response during ischemia and/or reperfusion in cardiac muscle (Xu et al. 1998; Zhu et al. 2007) and other organs (Koti et al. 2002; Mallick et al. 2005), potentially by increasing nitric oxide (NO) production. Importantly, an increase in NO levels induced by supplementation with NO metabolite compounds (e.g., L-arginine and nitrate) enhances exercise endurance and modifies systemic and local O_2_ response during exercise (Bailey et al. 2009, 2010; Larsen et al. 2011). Thus, one potential mechanism underlying IPC-induced effect may be the acceleration of systemic and local O_2_ dynamics due to NO-dependent mechanism. However, it remains unclear whether IPC ameliorates these O_2_ responses during exercise.

Previous studies have determined that the beneficial effects of IPC on exercise performance occurred independently of cardiac responses, as cardiac performance during exercise is not affected by IPC (de Groot et al. 2010; Crisafulli et al. 2011; Bailey et al. 2012a,b). Importantly, although a previous study reported that IPC can increase VO_2_ peak during a maximal ramp exercise test (de Groot et al. 2010), several other previous studies reported that IPC can increase the maximal exercise performance, not in VO_2_ peak (Crisafulli et al. 2011; Bailey et al. 2012a,b). Naturally, the VO_2_ peak is strongly affected by cardiovascular system in addition to peripheral system(s) (Åstrand et al. 1964), which suggests that IPC-induced improvement in exercise performance may result from peripheral adaptations, including those in the skeletal muscle rather than cardiovascular adaptation. Therefore, we proposed that the skeletal muscle adaptation, especially acceleration of muscle O_2_ response, may be necessary to achieve the beneficial effects of IPC on exercise performance.

Several recent studies have employed a unique work-to-work protocol that consists of double-step transitions from low- to moderate-intensity exercise and from moderate- to severe-intensity exercise (Williams et al. 2013; Da Boit et al. 2014). This protocol can help to observe the different modes of O_2_ response in a single exercise trial, and it can assess exercise endurance (i.e., the endurance time until exhaustion). Therefore, we hypothesized that IPC would enhance endurance exercise performance during a single work-to-work exercise, potentially by accelerating the muscular O_2_ dynamics. To test our hypothesis, we examined the effects of IPC on exercise endurance and the responses of pulmonary VO_2_ and muscle oxygenation during the work-to-work test.

## Methods

### Subjects

Fifteen habitually active, healthy male subjects (mean ± SE; age 24 ± 1 year, height 173 ± 1 cm, weight 68 ± 2 kg, systolic blood pressure 121 ± 2 mmHg) participated in this study. The subjects were informed about the experimental procedures and potential risks and gave written consent to participate in the study. All procedures were approved by the Ethics Committee of Ritsumeikan University (IRB-2013-050) and were conducted in accordance with the Declaration of Helsinki. Subjects were instructed to arrive at the laboratory in a rested and fully hydrated state, at least 4 h post prandial, and to avoid strenuous physical activity in the 24 h prior to each testing session. Each subject also abstained from caffeine and alcohol intake for 6 h and 24 h before each test, respectively. All tests were performed at the same time of day (±2 h).

### Experimental design

Subjects were required to visit the laboratory on three occasions over a 2-weeks period. At the first visit, subjects completed a ramp-incremental exercise test to determine the VO_2_ peak and gas exchange threshold (GET). Thereafter, the subjects were asked to perform two trials: one with and one without IPC. The order of trials were randomly assigned and counterbalanced. Since the previous study reported that the beneficial effects of IPC continue for at least 48 h in human (Loukogeorgakis et al. 2005), it was ensured that testing sessions were performed with an interval at least 1 week to eliminate the late effects of IPC. IPC consisted of three cycles of 5 min of bilateral arterial occlusion to the lower limbs, with 5 min of separation (i.e., the reperfusion period). The cuffs for the IPC were placed proximally around the upper thighs, whereas subjects were in a sitting position and were inflated to >300 mmHg. Previous studies have applied ischemic pressure for IPC at the absolute pressure of 220 mmHg (de Groot et al. 2010; Bailey et al. 2012a,b). In the preliminary testing, this study also employed the ischemic pressure of 220 mmHg. However, the use of this method caused gradual increases of values of total-hemoglobin/myoglobin (Hb/Mb), measured by near-infrared spectroscopy (NIRS), over a 5-min ischemia period. The result represents arterial blood inflow to the lower limbs, despite the exposure to ischemia. Hence, based on the results of several examinations, this study applied an ischemic pressure of >300 mmHg, and the application of this pressure did not cause the increase in total-Hb/Mb during the ischemia period. The control (CON) treatment followed the same experimental time (i.e., 30 min) without cuff inflation (de Groot et al. 2010; Crisafulli et al. 2011). The work-to-work exercise test started 5 min after the CON or IPC trials.

### Ramp-incremental test

At the first visit, all subjects performed a maximal incremental exercise test to determine the VO_2_ peak and GET on a cycling ergometer. Initially, subjects performed 3 min of baseline cycling at 30 W, after which the workload was increased at a rate of 30 W/min until the limit of tolerance. The subjects were asked to maintain a cadence of 60 rpm. The heights of saddle and bar in this test were recorded to reproduce the subsequent exercise tests. During the incremental test, breath-by-breath pulmonary gas exchange data were collected and averaged every 10 s. Heart rate (HR) was measured continuously via telemetry (RS400; Polar Electro Japan, Tokyo, Japan). The VO_2_ peak was determined as the highest 30-sec mean value attained prior to exhaustion. The exhaustion was assessed to be the maximum when three of the following criteria were obtained: (1) a plateau in the VO_2_ despite increasing workload (<100 mL/min), (2) a respiratory exchange ratio >1.10, (3) a heart rate within 10% of predicted maximal HR that was calculated as 220 – age, and (4) task failure of the pedaling rate of at least 55 rpm over 5 sec despite maximal effort. GET was determined using the V slope method, ventilatory equivalents, and end-tibial gas tension.

### Work-to-work exercise test

The data of VO_2_ peak and GET collected during the ramp-incremental test were used to calculate the work rate for moderate- and severe-intensity exercises. The work rate for both intensities employed 90% of the VO_2_ at GET (90% GET) and 70% of the difference between the VO_2_ at the GET and VO_2_ peak (70%Δ), respectively (Da Boit et al. 2014). Prior to experimental day, all the subjects familiarized themselves with the moderate-intensity and severe-intensity loads used during the test, which was performed after an analysis of the ramp-incremental test completed. During the work-to-work test, the first-step transition consisted of baseline low-intensity exercise at 30 W for 3 min followed by moderate-intensity exercise at 90% GET for 4 min. Next, the second step transitioned from a moderate- to severe-intensity exercise at 70%Δ. Thereafter, the subjects continued the severe-intensity exercise until exhaustion, which was defined by the criteria in the ramp-incremental test. The endurance time during severe-intensity exercise was evaluated as exercise endurance. In a pilot study, we assessed the test–retest reliability on two separate days with 10 young subjects, and the intraclass correlation coefficient (ICC) score of exercise endurance during severe-intensity exercise of the work-to-work test was 0.990. During the work-to-work exercise test, the pulmonary gas exchanges and NIRS-derived signals were continuously recorded to determine to the dynamics of the pulmonary VO_2_ and muscle oxygenation.

### Pulmonary VO_2_ dynamics

Pulmonary VO_2_ were measured by breath-by-breath using a gas analyzer (AE-310S; Minato Medical Science, Osaka, Japan). The breath-by-breath VO_2_ data collected during work-to-work exercise were edited by removing aberrant breath data (e.g., coughing, swallowing, sighing, etc.) that lay outside 4 SD of local mean. The VO_2_ data were interpolated to give second-by-second values and then were averaged into 5-sec bins. After this rectification, the VO_2_ data were used for determining the kinetics of pulmonary VO_2_ during a work-to-work exercise. To analyze the VO_2_ kinetics in moderate- and severe-intensity exercises, the first 20 sec of data during each step transition was deleted to remove the cardiodynamic response (i.e., phase I). A monoexponential model was used for moderate-intensity exercise, and a biexponential model was used for severe-intensity exercise, as described in the following equations: 

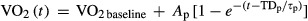
1

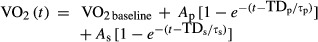
2where VO_2_ (*t*) represents VO_2_ at a given time *t*; VO_2 baseline_ is the mean VO_2_ in the baseline period; *A*_p_, TD_p_, and *τ*_p_ represent amplitude, time delay, and time constant, respectively, describing the primary response (i.e., phase II) in VO_2_ above baseline; and *A*_s_, TD_s_, and *τ*_s_ represent amplitude of, time delay before the onset of, and time constant describing the development of, the slow phase response (i.e., phase III), respectively. Baseline VO_2_ values in moderate- and severe-intensity exercises were defined as the mean VO_2_ measured over the final 60 sec during low- and moderate-intensity exercises, respectively. End-exercise VO_2_ value in moderate-intensity exercise was defined as the mean VO_2_ measured over the final 30-sec during exercise. The VO_2_ value at 240 sec after the onset of severe-intensity exercise was taken as the mean VO_2_ between 210 and 240 sec. End-exercise VO_2_ value in severe-intensity exercise was defined as the mean VO_2_ measured over 30 sec before the exhaustion in the exercise. The amplitude of VO_2_ during the slow phase response was described as the increase in VO_2_ from slow phase time delay to the end of exercise (Burnley et al. 2000). The mean response time, an overall kinetics, was calculated as the sum of the time delay and time constant in the primary response. In a pilot study, ICC scores of VO_2_ kinetics parameters during moderate- and severe-intensity phases of the work-to-work test were 0.978 and 0.975 for the time delay, 0.943 and 0.975 for the time constant, and 0.983 and 0.991 for the mean response time, respectively.

### Muscle oxygenation dynamics

Local tissue oxygenation profiles in the vastus lateralis (VL) muscle during exercise was measured using NIRS (NIRO 200; Hamamatsu Photonics, Shizuoka, Japan). During work-to-work exercise test, changes in oxy-Hb/Mb, deoxy-Hb/Mb, and total-Hb/Mb were sampled at 5 Hz and then averaged at 1 sec. This study predominantly analyzed the changes in deoxy-Hb/Mb, which is a marker of muscle deoxygenation. The muscle deoxygenation from deoxy-Hb/Mb is a reliable estimator of changes in intramuscular oxygenation states during exercise (De Blasi et al. 1994). The values of deoxy-Hb/Mb were calibrated using an arterial occlusion method (Hamaoka et al. 1996). To perform arterial occlusion, subjects completed a 3-min cool-down at 30 W after each exercise condition finished and then rested in a sitting position for 10 min. The arterial occlusion pressure was applied at the same pressure used for IPC. Arterial occlusion was continued for 10 min, at which time a maximal plateau value for deoxy-Hb/Mb was obtained for calibration. The baseline value for calibration was defined as the mean value measured over final 60 sec during low-intensity exercise in work-to-work test. The deoxy-Hb/Mb data during exercise were expressed as a percentage using baseline and maximal plateau values. To analyze the kinetics of deoxy-Hb/Mb during moderate- and severe-intensity exercises, deoxy-Hb/Mb were fitted with a monoexponential model in the following equation: 

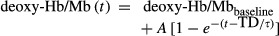
3where deoxy-Hb/Mb (*t*) represents deoxy-Hb/Mb at a given time *t*; deoxy-Hb/Mb _baseline_ is the baseline data before the onset of both step transients; *A*, TD, and *τ* represent amplitude, time delay, and time constant, respectively. This study employed a monoexponential model to determine the muscle deoxygenation dynamics in both exercise intensities (DeLorey et al. 2003). The time delays to an initial increase in deoxy-Hb/Mb after each step transition to moderate- and severe-intensity exercises were determined as the first point >1 SD above the mean of the baseline (DeLorey et al. 2003). Thereafter, deoxy-Hb/Mb was fitted from the time of initial increase to 120-sec during each intensity exercise. The basal information (e.g., amplitude) for deoxy-Hb/Mb was obtained with same time intervals to pulmonary VO_2_. The overall mean response time was calculated as the sum of the time delay and time constant. In a pilot study, ICC scores of the deoxy-Hb/Mb kinetics parameters during moderate- and severe-intensity phases of the work-to-work test were 0.883 and 0.897 for time delay, 0.910 and 0.973 for time constant, and 0.954 and 0.983 for the mean response time, respectively.

### Blood glucose and lactate

The fingertip blood sample was collected into a capillary tube to determine the blood glucose and lactate levels. A basal blood glucose level was measured to check for the fasting state before the CON or IPC trials on each testing day using a glucose analyzer (Glutest Neo Alpha; Sanwa Kagaku Kenkyusho, Nagoya, Japan). The fasting blood glucose level was equal on each testing day for the CON (93 ± 3 mg/mL) and IPC (97 ± 5 mg/mL).

The blood [lactate] levels were measured five times throughout the experiment using a lactate analyzer (Lactate Pro 2; Arkray, Kyoto, Japan). Blood sampling was performed as follows: before each trial (i.e., rest), 30 sec preceding the onset of the low-intensity exercise (i.e., pre-exercise), 30 sec preceding the transition to moderate-intensity exercise (i.e., end of low-intensity exercise), 30 sec preceding the transition to severe-intensity exercises (i.e., end of moderate-intensity exercise), and as soon as possible (<10 sec) after exhaustion due to severe-intensity exercise (i.e., exhaustion).

### Statistics

Statistical analysis for comparisons between CON and IPC was performed using a paired Student's *t*-test. The level of significance was set at *P *< 0.05. All statistical tests were performed using SPSS 16.0 (International Business Machines Corporation, NY, USA).

## Results

Mean values of VO_2_ peak and GET, as measured by the ramp-incremental test in the subjects, were 3.19 ± 0.13 L/min and 1.98 ± 0.11 L/min, respectively. On the basis of GET and VO_2_ peak, the mean workloads of the moderate- and severe-intensity exercises in the work-to-work test were 138 ± 3 W and 233 ± 7 W, respectively.

**Figure 1:**
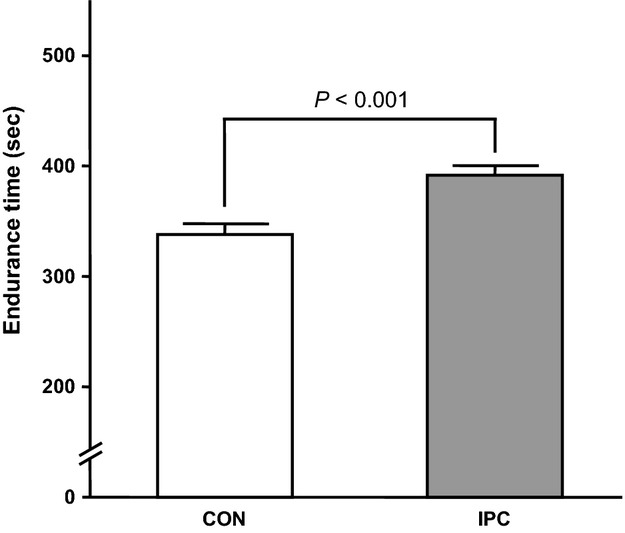
Endurance time to exhaustion during severe-intensity exercise in the work-to-work cycling exercise test. Values are presented as mean ± SE.

**Figure 2:**
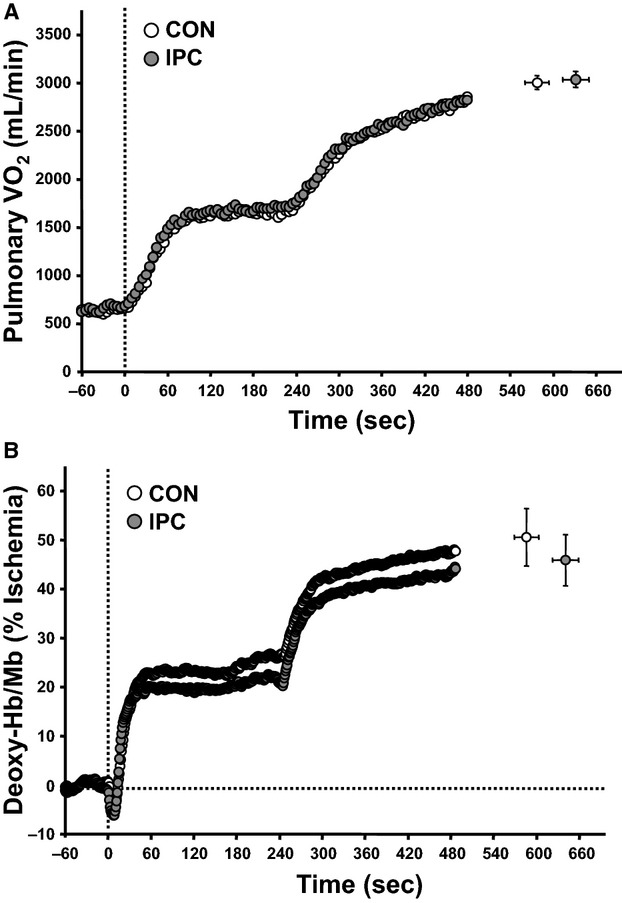
Pulmonary oxygen uptake (VO_2_) and deoxy-Hb/Mb responses during work-to-work exercise. Panel A illustrates the pulmonary VO_2_ responses during work-to-work exercise. Data are shown as 5-sec mean values after rectification. Panel B illustrates the deoxy-Hb/Mb responses during work-to-work exercise. Data are expressed as a percentage of the highest plateau value during 10-min arterial occlusion. The systemic and local O_2_ responses following the CON and IPC trials are presented as open and solid circles, respectively. The dotted vertical line at zero in panels A and B indicates the transition from low-intensity exercise to moderate-intensity exercise. The dotted horizontal line in panel B indicates the baseline in deoxy-Hb/Mb. End-exercise values for pulmonary VO_2_ and deoxy-Hb/Mb are presented as mean ± SE.

The result of exercise endurance is shown in Figure1. As expected, exercise endurance (i.e., the time to exhaustion in severe-intensity exercise) was significantly greater in IPC than in CON. Results of the dynamics of pulmonary VO_2_ during the work-to-work cycling exercise test are shown in Table1 and Figure2. Mean values of pulmonary VO_2_ at the end-exercise of the work-to-work exercise in CON and IPC conditions reached 95.4 ± 4.2% and 97.0 ± 4.2%, respectively, suggesting that volitional fatigue was achieved in both conditions. Alternatively, changes in the pulmonary VO_2_ throughout the work-to-work test were not significantly different between CON and IPC conditions. Moreover, the kinetics of pulmonary VO_2_ during the moderate- and severe-intensity exercises were not significantly different between the two trials.

**Table 1:** Kinetics parameters of pulmonary oxygen uptake during work-to-work exercise

	CON	IPC
Moderate-intensity exercise		
Baseline, L/min	0.64 ± 0.02	0.67 ± 0.02
End-exercise, L/min	1.66 ± 0.03	1.73 ± 0.03
Primary amplitude, L/min	1.02 ± 0.03	1.07 ± 0.03
Primary time delay, sec	22.0 ± 1.7	20.3 ± 1.3
Primary time constant, sec	30.6 ± 3.1	31.7 ± 2.8
Mean response time, sec	52.6 ± 4.6	52.0 ± 3.9
Severe-intensity exercise		
Baseline, L/min	1.66 ± 0.03	1.65 ± 0.03
Primary amplitude, L/min	0.89 ± 0.05	0.93 ± 0.06
240 sec, L/min	2.80 ± 0.06	2.79 ± 0.06
Primary time delay, sec	36.3 ± 4.6	31.2 ± 4.1
Primary time constant, sec	64.9 ± 6.2	54.3 ± 7.3
Mean response time, sec	101.2 ± 9.3	85.5 ± 11.0
Slow phase time delay sec	138.7 ± 8.1	146.0 ± 7.0
Slow phase amplitude, L/min	0.43 ± 0.03	0.44 ± 0.05
End-exercise, L/min	2.97 ± 0.07	3.03 ± 0.08

Values are presented as mean ± SE.

**Figure 3:**
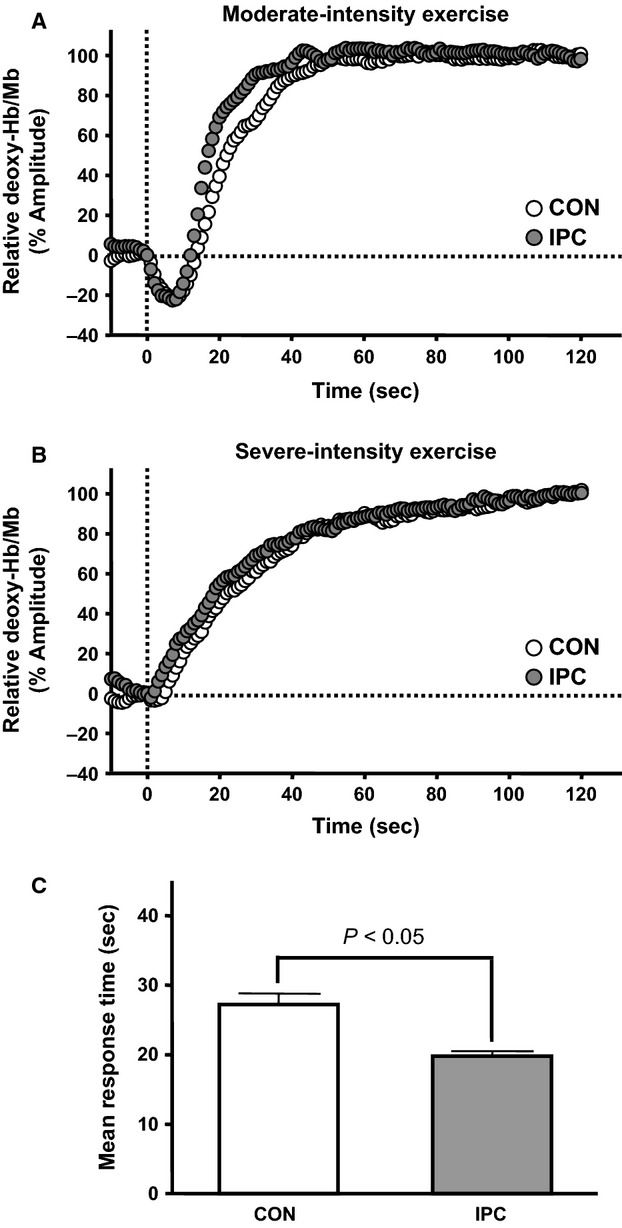
Muscle deoxygenation responses during moderate- and severe-intensity exercises in work-to-work exercise. Panels A and B illustrate the relative changes of deoxy-Hb/Mb during moderate- and severe-intensity exercises, respectively. Data are normalized relative to the end-exercise amplitude. Panel C shows the mean response time of muscle deoxygenation during moderate-intensity exercise. Values are presented as mean ± SE.

The results of dynamics of deoxy-Hb/Mb are shown in Table2, Figures2 and 3. The amplitude of deoxy-Hb/Mb during moderate-intensity exercise was significantly smaller in IPC than in CON. However, the same trend was not observed during severe-intensity exercise. The time delay and time constant for muscle deoxygenation after the transition from low- to moderate-intensity exercise tended to be shorter in IPC than in CON, although the difference was not statistically significant. More importantly, the mean response time for muscle deoxygenation during moderate-intensity exercise was significantly shorter in IPC than in CON. In contrast, the kinetics of the transition in severe-intensity exercise were unaffected by IPC.

**Table 2:** Kinetics parameters of deoxy-hemoglobin/myoglobin (Hb/Mb) during work-to-work exercise

	CON	IPC
Moderate-intensity exercise		
Amplitude, %	27.3 ± 4.1	21.4 ± 3.5*
Time delay, sec	14.8 ± 1.6	11.9 ± 0.7
Time constant, sec	12.4 ± 2.2	7.8 ± 1.0
Mean response time, sec	27.2 ± 2.9	19.8 ± 0.9*
Severe-intensity exercise		
Baseline, %	26.7 ± 4.0	21.2 ± 3.4*
240 sec, %	49.3 ± 5.6	42.8 ± 4.6
End-exercise, %	51.6 ± 5.8	45.6 ± 4.9
Amplitude, %	24.9 ± 3.5	24.4 ± 3.0
Time delay, sec	7.2 ± 1.8	5.3 ± 1.6
Time constant	24.4 ± 4.3	29.7 ± 4.7
Mean response time, sec	31.6 ± 4.5	35.1 ± 4.8

Values are presented as mean ± SE. The values for deoxy-Hb/Mb are expressed as a percentage of the highest plateau value during 10-min arterial occlusion.

* Significant difference from the CON trial: *P *< 0.05.

Results of the heart rate and blood [lactate] responses are shown in Table3. There was no significant difference in the changes in HR throughout the exercise between CON and IPC. Additionally, no significant difference was observed in the changes in the blood lactate level during exercise between both trials.

**Table 3:** Heart rate and blood [lactate] responses during work-to-work exercise

	CON	IPC
Heart rate, beats/min		
Rest	68 ± 3	67 ± 2
Pre-exercise	69 ± 2	64 ± 4
End of low-intensity exercise	81 ± 2	81 ± 1
End of moderate-intensity exercise	127 ± 4	127 ± 2
Exhaustion	179 ± 4	180 ± 3
Blood [lactate], mM		
Rest	1.1 ± 0.1	1.1 ± 0.1
Pre-exercise	1.3 ± 0.2	1.4 ± 0.1
End of low-intensity exercise	1.8 ± 0.3	2.1 ± 0.2
End of moderate-intensity exercise	3.7 ± 0.3	3.8 ± 0.4
Exhaustion	13.6 ± 0.9	13.8 ± 1.3
Δblood [lactate]	12.2 ± 0.9	12.4 ± 1.2

Values are presented as mean ± SE. Rest, before each trial; Pre-exercise, 30 sec preceding the onset of the low-intensity exercise; End of low-intensity exercise, 30 sec preceding the transition to moderate-intensity exercise, End of moderate-intensity exercise, 30 sec preceding the transition to severe-intensity exercises; Exhaustion, as soon as possible (<10 sec) after exhaustion from severe-intensity exercise; [Lactate], lactate concentration; Δ, change between pre-exercise and exhaustion.

## Discussion

The principal findings in this study were that IPC accelerated muscle deoxygenation in moderate-intensity exercise and enhanced severe-intensity exercise endurance during a work-to-work cycling exercise. Previous studies have reported that IPC enhances maximal exercise performance, such as VO_2_ peak and maximal workload (de Groot et al. 2010; Crisafulli et al. 2011). The present findings extended these previous findings by showing that IPC can enhance submaximal endurance exercise performance. Alternatively, our findings showed that the dynamics of pulmonary VO_2_ during the exercise was unaffected by IPC. Moreover, the VO_2_ values at exhaustion during severe-intensity exercise did not increase because of IPC treatment. A previous study reported that the VO_2_ peak during the maximal exercise test was increased by IPC (de Groot et al. 2010). In contrast, several other previous studies showed that IPC increased the maximal workload during the incremental ramp exercise test, but it did not affect the VO_2_ peak (Crisafulli et al. 2011; Bailey et al. 2012a,b). Although the integrity of the effect of IPC on the pulmonary VO_2_ response during exercise remains unknown, the present findings may support the fact that IPC has no impact on systemic O_2_ responses during exercise. Pulmonary O_2_ dynamics are mainly affected by cardiovascular and skeletal muscle responses. For example, a faster induction of pulmonary O_2_ dynamics induced by prior exercise results from cardiovascular activation in addition to skeletal muscle activation (Burnley et al. 2000; Gurd et al. 2005; Marles et al. 2007; Faisal et al. 2009). Compared with prior exercise, IPC did not have a beneficial effect on the cardiovascular response during exercise (de Groot et al. 2010; Crisafulli et al. 2011; Bailey et al. 2012a,b). In this study, the changes in HR during exercise was also unaffected by IPC. Therefore, these findings suggest that the effect of IPC on exercise performance may be achieved by the adaptation of local skeletal muscle rather than the systemic cardiovascular system.

To our knowledge, this study is the first to determine that IPC accelerates the muscular O_2_ response during systemic whole-body exercise. We evaluated muscle deoxygenation using NIRS-derived deoxy-Hb/Mb. A part of the NIRS signal parameters indicate muscular O_2_ extraction (DeLorey et al. 2003), which reflects the regional balance between O_2_ utilization and O_2_ availability. Therefore, the observed IPC-induced acceleration of muscle oxygenation dynamics may result from accelerated O_2_ extraction in skeletal muscle. Previous studies have shown that prior muscle contractions in rodent and *Xenopus* muscles can speed up muscle vascular O_2_ dynamics during subsequent muscle contractions (Hogan 2001; Behnke et al. 2002). The findings of these previous models clearly showed that prior muscle contraction induces an accelerated muscle O_2_ response, suggesting that IPC may be able to achieve a beneficial effect similar to prior exercise treatment without muscle contractions. Moreover, prior exercise-induced acceleration of the muscle O_2_ response is associated with mitochondrial enzymatic activation in skeletal muscle (Gandra et al. 2012). The mitochondrial enzyme activity in skeletal muscle is acutely enhanced by a single bout of exercise (Leek et al. 2001; Fernström et al. 2004), and it may be related to the accelerated O_2_ metabolism. Therefore, we propose that IPC enhances exercise performance with skeletal muscle mitochondrial activation, as indicated by the accelerated O_2_ extraction.

In previous studies, potential molecule(s) underlying IPC-induced enhancement of exercise performance remain unknown. It is well known that NO is produced from vascular endothelial cells following an increase in shear stress (Kooijman et al. 2008; Green et al. 2014), which is induced by the raid increase in blood flow, such as that occurs with reperfusion of IPC. In fact, a recent study determined that IPC increased the levels of the blood NO metabolites in human (Rassaf et al. 2014). The study also clearly showed that the beneficial effects of IPC are strongly regulated by NO produced from endothelial NO synthase in mice. Previous studies have reported that supplementation with NO metabolite compounds improves exercise performance (Bailey et al. 2009, 2010; Larsen et al. 2011). Moreover, enhanced NO levels reduce the local muscular O_2_ cost in addition to the systemic O_2_ cost during exercise (Bailey et al. 2009, 2010), by improving energy efficiency in the mitochondria of the skeletal muscle (Larsen et al. 2011). In this study, IPC did not affect the systemic pulmonary O_2_ cost during the work-to-work exercise test. In contrast, IPC reduced the amplitude of deoxy-Hb/Mb during moderate-intensity exercise, but not during severe-intensity exercise. This finding suggests that IPC may decrease the O_2_ cost in moderate exercising muscles, which is consistent with the beneficial effects of supplementation with NO metabolite compounds. Importantly, Bailey et al. (2012a,b) demonstrated that IPC enhanced running performance during strenuous exercise and prevented flow-mediated dilation (FMD)-measured endothelial dysfunction after this exercise, suggesting that IPC-induced enhancement of exercise performance may be related to NO bioavailability. Therefore, we suggest that one potential molecule underlying IPC-induced effects may result from increased NO synthase.

This study has several limitations. First, this study finding showed that dynamics of deoxygenation in skeletal muscle during moderate-intensity in the work-to-work test was accelerated by IPC; however, this was not observed during severe-intensity exercise. It is difficult to explain the discrepancy between the moderate- and severe-intensity phases from only the present findings. Nevertheless, in a recent study, Patterson et al. (in press^2015^) reported that IPC enhanced the power output during the early phase in repeated sprint cycling exercise, which suggests that IPC-induced effects on exercise performance may be dissolved over the time course from IPC. In fact, the response in FMD during reperfusion, which is induced by increasing NO production, reverses within 5 min (Nishiyama et al. 2007). Hence, a benefit from IPC may disappear until the time point (i.e., 12 min after the last ischemia has finished) of the transition from moderate- to severe-intensity; however, the advantage during moderate-intensity exercise may be useful enough for enhancing severe-intensity exercise endurance. Alternatively, in contrast to our speculation, Bailey et al. (Bailey et al. 2012a,b) reported that IPC-induced enhancement of exercise performance was observed over 1 h after IPC. Thus, further study would be needed to examine the duration of IPC-induced effect.

Next, previous studies analyzed the dynamics of pulmonary VO_2_ and muscle deoxygenation by measuring several bouts over a short-term period (DeLorey et al. 2003; Bailey et al. 2010), and they were averaged to rectify the signal-to-noise ratio. However, this study analyzed the dynamics of pulmonary VO_2_ and muscle deoxygenation by using only a single bout of a work-to-work test. Previous studies determined that IPC provides protection from tissue damage for up to 24 h in rodents (Marber et al. 1993; Xuan et al. 2007). Moreover, a previous study reported that the beneficial effects of IPC continue for at least 48 h in human (Loukogeorgakis et al. 2005). As the length of the late effects of IPC remains unclear, each testing session was separated by an interval of at least 1 week to eliminate bias. Thus, a relatively long period is required when performing multiple measurements; however, it is not easy to control the physiological states of the subjects. In addition, the analysis of the dynamics of pulmonary VO_2_ and muscle deoxygenation has been performed in many previous studies not only using multiple bouts but also a single bout (Wilkerson et al. 2004). In a previous pilot study, we examined test–retest reliability of systemic and local O_2_ dynamics during the work-to-work test, and the ICC showed a reasonably high value. Thus, we believe that IPC-induced acceleration of muscle deoxygenation dynamics during the exercise is reliable.

In conclusion, the findings of this study showed that IPC accelerated muscle deoxygenation dynamics in moderate-intensity exercise and endurance in severe-intensity exercise during a work-to-work test. The IPC-induced effects may result from mitochondrial activation in skeletal muscle, as indicated by the accelerated O_2_ extraction, which may have the enhanced NO synthase. Further study is needed to clarify the biological and molecular mechanisms underlying the effects of IPC on exercise performance.
